# A platform for locoregional T-cell immunotherapy to control HNSCC recurrence following tumor resection

**DOI:** 10.18632/oncotarget.27982

**Published:** 2021-06-22

**Authors:** Shay Sharon, Jason R. Baird, Shelly Bambina, Gwen Kramer, Tiffany C. Blair, Shawn M. Jensen, Rom S. Leidner, R. Bryan Bell, Nardy Casap, Marka R. Crittenden, Michael J. Gough

**Affiliations:** ^1^Department of Oral and Maxillofacial Surgery, Hadassah and Hebrew University Medical Center, Jerusalem 9112001, Israel; ^2^Earle A. Chiles Research Institute, Robert W. Franz Cancer Center, Providence Portland Medical Center, Portland, OR 97213, USA; ^3^Department of Molecular Microbiology and Immunology, Oregon Health and Sciences University, Portland, OR 97239, USA; ^4^The Oregon Clinic, Portland, OR 97213, USA

**Keywords:** head and neck cancer, T-cell, immunotherapy, biomaterial, intratumoral

## Abstract

Surgical resection of head and neck squamous-cell carcinoma (HNSCC) is associated with high rates of local and distant recurrence, partially mitigated by adjuvant therapy. A pre-existing immune response in the patient’s tumor is associated with better outcomes following treatment with conventional therapies, but improved options are needed for patients with poor anti-tumor immunity. We hypothesized that local delivery of tumor antigen-specific T-cells into the resection cavity following surgery would direct T-cells to residual antigens in the margins and draining lymphatics and present a platform for T-cell-targeted immunotherapy. We loaded T-cells into a biomaterial that conformed to the resection cavity and demonstrated that it could release T-cells that retained their functional activity *in-vitro*, and in a HNSCC model *in-vivo*. Locally delivered T-cells loaded in a biomaterial were equivalent in control of established tumors to intravenous adoptive T-cell transfer, and resulted in the systemic circulation of tumor antigen-specific T-cells as well as local accumulation in the tumor. We demonstrate that adjuvant therapy with anti-PD1 following surgical resection was ineffective unless combined with local delivery of T-cells. These data demonstrate that local delivery of tumor-specific T-cells is an efficient option to convert tumors that are unresponsive to checkpoint inhibitors to permit tumor cures.

## INTRODUCTION

Tumor-positive surgical margins are one of the most important risk factors for recurrence following surgical resection of HNSCC. Local recurrence develops even in patients who have histologically negative margins, and occurs at a high rate in patients with intermediate and high-risk features [1]. This observation suggests that residual tumor cells are often present following surgery, which leads to locoregional recurrence despite clear margins.

Adjuvant chemo/radiotherapy represents a partial solution to this problem [2], but there may also be a role for immunotherapy which is recently being evaluated in the neoadjuvant setting [3, 4]. Enhancing tumor-specific immune responses at the time of the initial procedure will be valuable for control of residual microscopic disease, and to direct immune cells against distant tumor deposits [5]. Adoptive therapy of tumor antigen-specific T-cells is a proven approach to control metastatic disease in selected patients, and is generally delivered intravenously. Following adoptive transfer, T-cell expansion and *in-vivo* differentiation into tumoricidal effector cells is essential for effective therapy [6–8]. Secondary proliferation of transferred T-cells occurs in tumor-draining lymph nodes [7], meaning that adoptively-transferred cells are dependent on ongoing tumor antigen cross-presentation for full functional capacity.

These data present a significant problem in the use of adoptive T-cell therapy for high-risk patients to treat minimal residual disease following surgery. In this scenario, there may be a narrow timeframe following surgical resection to successfully expand T-cells *in-vitro*, before cross-presented antigen is lost from the tumor-draining lymph node [9]. Therefore, it would potentially be advantageous to rapidly direct transferred T-cells to the tumor-draining lymph node for *in-vivo* expansion, before cross-presentation of tumor-derived material is lost. One approach to achieve this is to load the T-cells into the tumor-draining lymphatics via local delivery, to give the maximum chance of meeting cognate antigen on antigen-presenting cells to permit *in-vivo* expansion. By contrast, systemically applied T-cells are likely to evenly distribute between tissues and secondary lymphoid organs throughout the patient while their target antigen is restricted to a specific lymphatic drainage system. This may result in an immediate reduction in the proportion of transferred cells that can meet their cognate antigen in the narrow time window [10].

The tumor resection cavity can be a poorly defined and disrupted site for the local delivery of cells and drugs. Biomaterials can serve as a vehicle to physically support agents in the resection cavity and permit locoregional delivery [11] of innate adjuvants in preclinical models of HNSCC [12] and other cancers [13]. Importantly, T-cells can be incorporated into biomaterials for locoregional delivery [14, 15], and combined with additional T-cell activating agents to improve tumor control [16, 17]. We propose that biomaterial delivery of T-cells to the resection site will naturally deliver these cells into contact with residual cancer cells or into the lymphatic drainage for expansion and recirculation to prevent local recurrence. We demonstrate in preclinical models that biomaterial-loading preserves T-cell function *in-vitro* and *in-vivo*, and in a surgical model of HNSCC results in control of tumor recurrence. Importantly, we show that adjuvant therapy with anti-PD1 fails to impact local recurrence unless combined with local delivery of tumor-specific T-cells, demonstrating that this approach has the potential to help patients with poor pre-existing anti-tumor reactivity. Together, these data demonstrate relevant locoregional immunotherapy with the potential to overcome locoregional recurrence for the benefit of high-risk HNSCC patients, and integration with conventional therapies.

## RESULTS

### *In-vitro* characterization of T-cells loaded in a biomaterial

**Figure 1:**
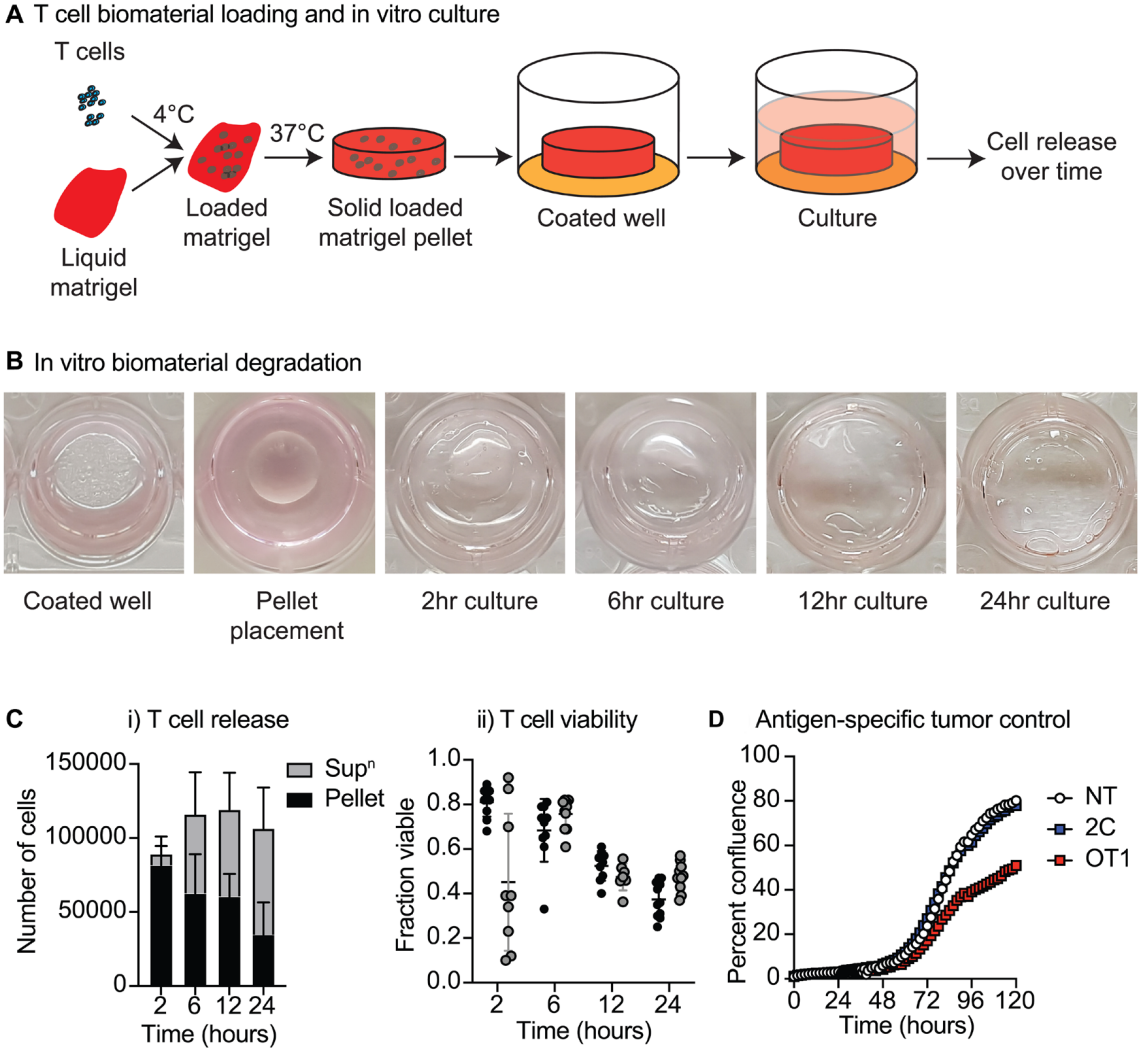
Biomaterial loading of T-cells. (**A**) Matrigel pellets containing purified CD8 T-cells from naïve C57BL/6 mice were seeded to 24 well Matrigel-coated plates and covered with media. (**B**) Images show placement and degradation of the pellet over time. (**C**) Culture supernatant and Matrigel were isolated over time and the number of viable T-cells in each compartment weredetermined. Graphs show (i) number of T-cells in supernatant and pellet over time, (ii) viability of T-cells in the supernatant and pellet over time. (**D**) Functional response of loaded T-cells. The well was coated with Moc1 cells engineered to express the model antigen SIINFEKL (Moc1-ova), and the Matrigel pellet was loaded with OT1 T-cells specific for SIINFEKL, 2C T cells with irrelevant specificity, or no T-cells (NT). Graph shows confluency of cancer cells over time.

Matrigel represents a convenient laboratory hydrogel that is a liquid when chilled, but solidifies into an amorphous solid at body temperature. This permits rapid local conformation of the loaded biomaterial to the unique shape of the resection cavity following surgery [12]. To function as a T-cell delivery system, it is critical that loaded T-cells retain their function over the course of biomaterial degradation and cell release. To study the dynamics of T-cells loaded into a biomaterial, CD8^+^ T-cells were isolated from naïve C57/BL6 mice and introduced into Matrigel to form pellets (Figure 1A). After 2, 6, 12 and 24 hours the culture supernatant and Matrigel were isolated and the number of viable T-cells in each compartment was determined. The T-cell pellet rapidly degraded in culture (Figure 1B), and analysis of the pellet and supernatant revealed that the number of T-cells in supernatant increased over time while the number of T-cells in the pellet decreased over time, suggesting the sustained release of T-cells from the biomaterial during this timeframe (Figure 1Ci). Despite the absence of antigen or T-cell growth factors, there was no consistent difference in viability between T-cells in the biomaterial or in the culture supernatant (Figure 1Cii). To test the functionality of the released T-cells, we used an assay that could assess control of cancer cell growth by T-cells being released from the pellets over time. We coated wells with Moc1-ova cells engineered to express the model antigen SIINFEKL as a fusion protein to ensure non-secreted cytoplasmic protein expression (Moc1-ova). These cells were later overlaid with a Matrigel pellet that was loaded with OT1 cells specific for SIINFEKL, 2C T-cells with irrelevant specificity, or no T-cells. Wells were imaged at 2-hour intervals, and the confluency of the cancer cells in replicate wells was determined as previously described [18]. OT1 cells loaded into a biomaterial significantly decreased the confluency of Moc1-ova cells compared to 2C T-cells, or unloaded biomaterial (*p* < 0.01) (Figure 1D). Together, these data demonstrate that T-cells loaded into Matrigel undergo a sustained release *in-vitro* and are functionally active against cancer cells expressing their target antigen.

### Tumor control following intratumoral injection of antigen-specific T-cells

**Figure 2:**
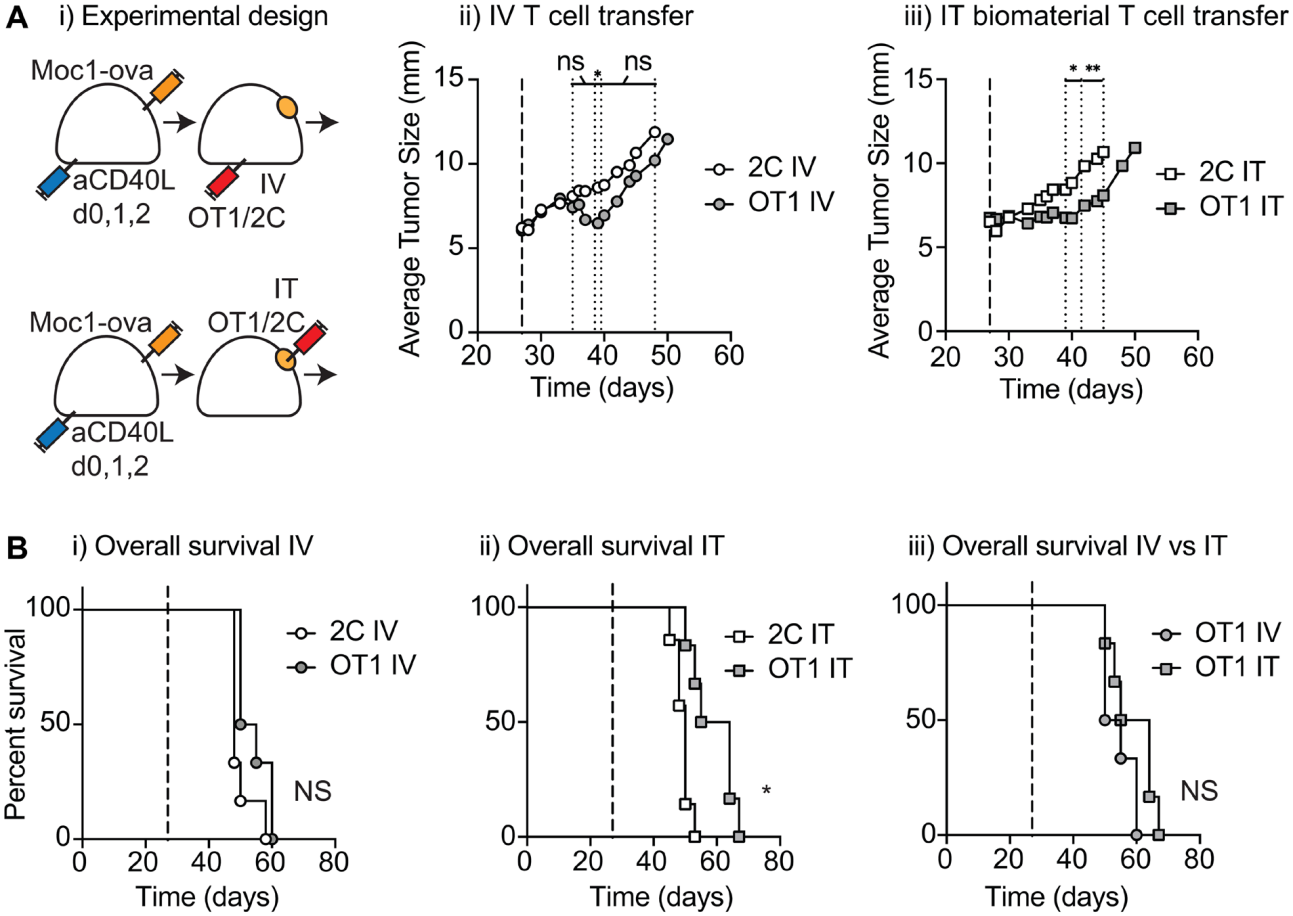
Tumor control following injection of antigen-specific T cell biomaterial. (**A**) (i) MOC1-ova tumors were established in immune competent C57BL/6 mice along with anti-CD40L to block implantation-related immune responses, and randomized to receive intravenous (IV) or intratumoral (IT) adoptive T cell transfer to established tumors. Mice received 1 × 10^6^ tumor-specific OT1 T-cells or non-specific 2C T-cells IV in suspension, or in 30 μl of Matrigel into the tumor (IT). Graphs show (ii) tumor growth following IV transfer, or (iii) tumor growth following IT transfer. (**B**) Overall survival of groups treated as in (A), showing (i) IV treatment groups, and (ii) IT treatment groups. (iii) comparison of overall survival of mice treated with OT1 T cells IV versus IT. Experiments incorporated 6–8 mice per group and the displayed experiment is representative of 3 independent repeats. Abbreviation: NS: not significant. ^*^ *p* < 0.05, ^**^ *p* < 0.01.

To determine whether biomaterial loaded T-cells would control a tumor *in-vivo*, we evaluated direct intratumoral (IT) delivery. As a comparison to conventional adoptive transfer approaches, we also evaluated the systemic delivery of T-cells alone via IV injection. Moc1-ova tumors were established in immune-competent C57BL/6 mice that were also injected with three doses of anti-CD40L, which blocks the anti-tumor immune response following tumor implantation (not shown). This approach results in tumors that lack pre-existing anti-tumor immunity and are poorly responsive to conventional therapies [19], and is a model for patients who may most benefit from the transfer of tumor-specific T-cells. Mice were randomized to receive IV or IT adoptive T-cell transfer, and as a control for the invasiveness of intratumoral injection, mice were randomized to receive tumor antigen-specific OT1 T-cells or non-specific 2C T-cells. In this way, the tumors treated with OT1 versus 2C cells differ only in the TCR of the transferred cells, providing a highly relevant control treatment. 1 × 10^6^ tumor-specific OT1 or non-specific 2C T-cells were administered intravenously in suspension, or loaded into Matrigel and injected into the tumor (Figure 2Ai). IT administration of OT1 T-cell-loaded biomaterial resulted in tumor growth delay compared to IT administration of 2C T-cell controls, demonstrating that T-cell function was retained *in-vivo* (Figure 2Aiii). IV administration of OT1 T-cells also resulted in transient tumor growth delay (Figure 2Aii); neither approach was curative, and there was not a significant difference in overall survival between OT1 T-cells administered via IV versus IT transfer (Figure 2B), suggesting that local biomaterial delivery was equivalent in efficacy to conventional IV transfer. These findings underscore the potential of local delivery of T-cells into the tumor, achieving local control rates that are equivalent to the corresponding intravenous therapy.

### Characterization and phenotyping of circulating T-cells following adoptive T-cells transfer

**Figure 3:**
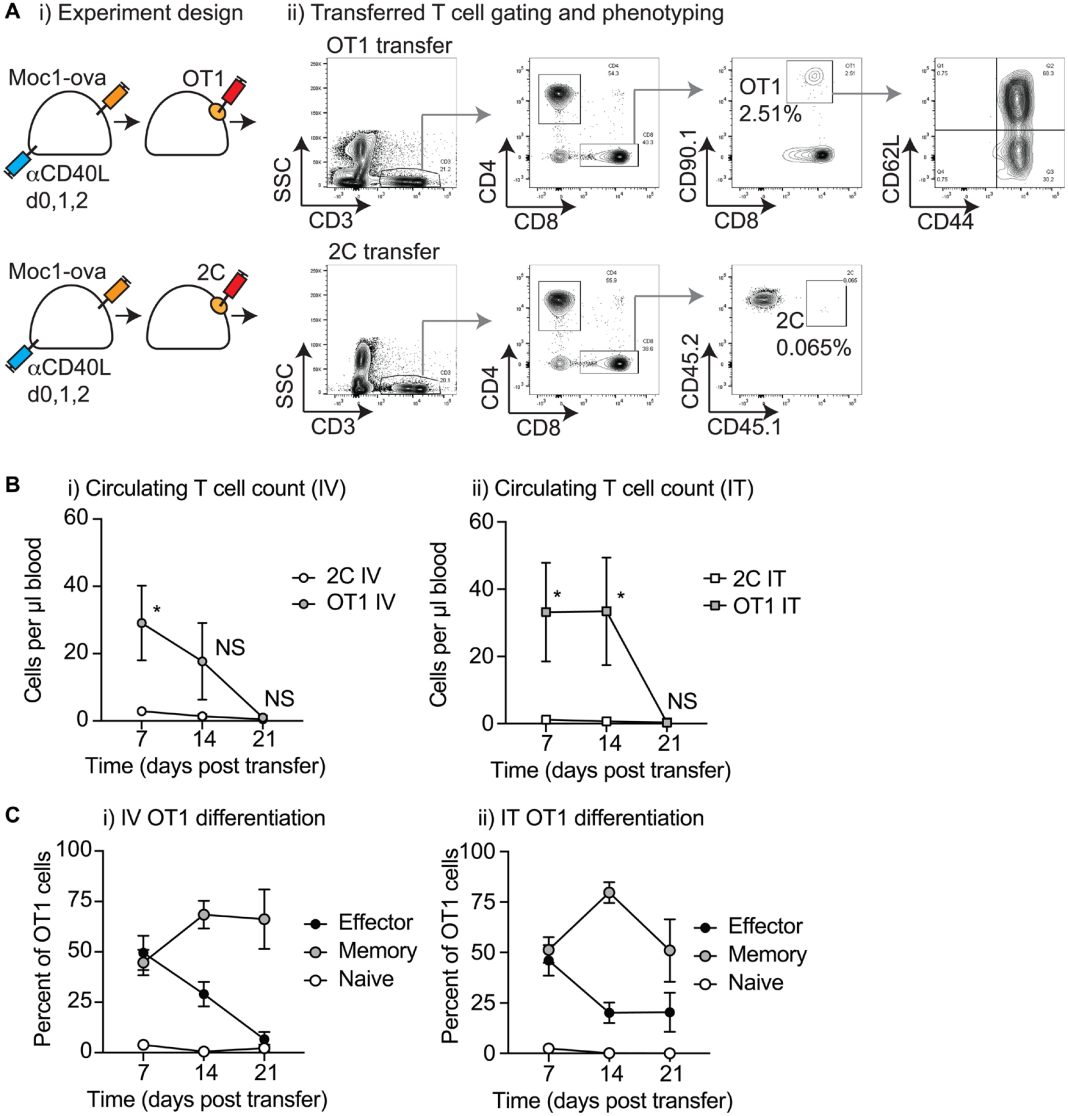
Characterization of circulating T-cells following biomaterial injection. (**A**) (i) MOC1-ova tumors were established in immune competent C57BL/6 mice and randomized to receive intravenous (IV) or intratumoral (IT) adoptive T-cell transfer. Mice received 1 × 10^6^ tumor-specific OT1 T-cells or non-specific 2C T-cells IV in suspension, or in 30 μl of Matrigel into the tumor (IT). (ii) Representative flow cytometry plots show whole blood 14 days following adoptive T-cell transfer showing identification of CD3+CD8+CD90.1+ OT1 T cells or CD3+CD8+CD45.2+ 2C T-cells. (**B**) Quantitative analysis of the number of OT1 T-cells and 2C T-cells in the peripheral blood over time following transfer (i) IV as a suspension or (ii) IT as a biomaterial. Phenotypic analysis of Effector (CD44+CD62L-, Memory (CD44+CD62L+), or Naive (CD44-CD62L+) among gated OT1 T-cells over time following transfer (i) IV as a suspension, or (ii) IT as a biomaterial. (**C**) Phenotypic analysis of Effector (CD44+CD62L–, Memory (CD44+CD62L+), or Naive (CD44–CD62L+) among gated OT1 T-cells over time following transfer (i) IV as a suspension, or (ii) IT as a biomaterial. Abbreviation: NS: not significant, ^*^ *p* < 0.05.

To evaluate whether IT transfer resulted in effective release and recirculation of tumor antigen-specific T-cells, we examined peripheral blood of treated animals to identify the number and phenotype of the transferred T-cells (Figure 3Ai). Adoptively transferred T-cells could be distinguished by congenic markers, allowing the identification of circulating CD3^+^CD8^+^CD90.1^+^ OT1 T-cells and CD3^+^CD8^+^CD45.1^+^ 2C T-cells (Figure 3Aii). As expected, tumor antigen-specific OT1 T-cells were present in significantly higher numbers than non-specific 2C T-cells, and the OT1 T-cells declined in number over time (Figure 3B). Loss of these cells from the peripheral circulation broadly correlated with tumor progression, where tumors in all OT1 transferred mice had resumed growth over the 14–21 days period following transfer (Figure 2). To determine whether local delivery altered the differentiation of T-cells, we evaluated the expression of CD44 and CD62L tumor antigen-specific T-cells to identify differentiation into CD44^+^CD62^-^ effector, CD44^+^CD62L^+^ memory, or CD44^-^CD62L^+^ naïve T-cells. Following both IV and IT treatment, both effector and memory populations were observed; however, effector cells were more rapidly lost from peripheral circulation and the small residual circulating population at later time-points was mostly memory T-cells (Figure 3C). There was not a significant difference in the number or phenotype of OT1 T-cells that had been delivered via biomaterial injection into the tumor versus conventional IV delivery, suggesting that local delivery into a tumor environment does not impair T-cell function.

### Characterization of the tumor environment following injection of T-cell biomaterial

**Figure 4:**
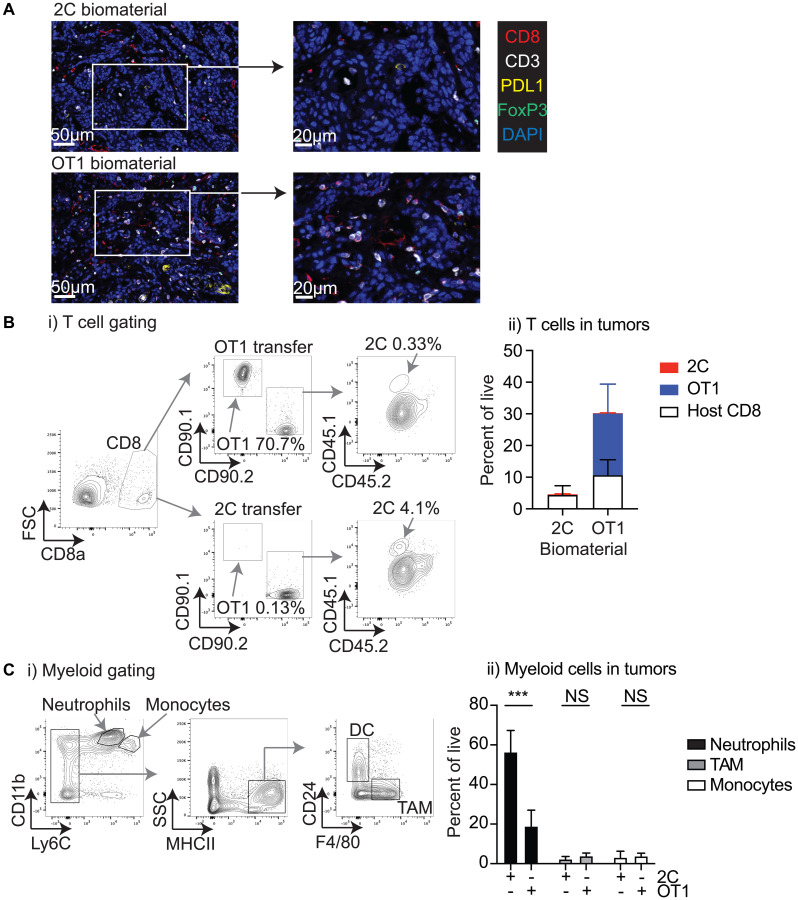
Characterization of the tumor environment following biomaterial injection. MOC1-ova tumors were established in immune competent C57BL/6 mice and randomized to receive 1 × 10^6^ tumor-specific OT1 T-cells or non-specific 2C T-cells in 30 μl of Matrigel into the tumor. Tumors were harvested 7 days later for analysis of infiltrating immune cells. (**A**) Representative images following multiplex IHC for infiltrating immune cells in the tumor following injection of 2C or OT1 biomaterial. (**B**) (i) Identification of congenic CD90.1+CD90.2- OT1 and CD90.2+CD45.1+ 2C in the tumor from infiltrating CD8 T-cells. (ii) Graph of mean and standard deviation of infiltrating CD8 T-cells and the proportion that are host, transferred OT1, or transferred 2C. (**C**) (i) Representative flow cytometry plots show gating for major myeloid populations in the tumor. (ii) Proportions of Neutrophils, TAM, and monocytes in tumors treated with 2C or OT1 biomaterials Abbreviation: NS: not significant. ^*^ *p* < 0.05, ^***^ *p* < 0.001.

To determine whether biomaterial delivery resulted in functional T-cell accumulation in the tumor, we examined the tumor immune environment 7 days following IT treatment, at the peak of tumor control. Tumors were harvested, bisected, and analyzed in parallel for T-cell infiltration by multiplex IHC and flow-cytometry. IHC analysis demonstrated that tumors treated with OT1-loaded biomaterials exhibited a less defined organization compared to the more clearly defined structure of the 2C specimens (Figure 4A). Notably, in the OT1-injected samples, CD3^+^CD8^+^ cells were located throughout the tumor, while CD3^+^CD8^+^ cells in the 2C-injected samples were mostly confined to stromal regions. Quantitative analysis showed CD3^+^CD8^+^ T-cells were more frequent in the OT1-injected samples (data not shown). FoxP3^+^ and PD-L1^+^ cells did not differ substantially between the groups, as determined by multiplex IHC (Figure 4A). IHC analysis was not able to distinguish transferred T-cells from host T-cells in the tumor, so the matching specimens were analyzed by flow-cytometry for the transferred cells. As with the peripheral blood, infiltrating transferred cells were distinguished using congenic markers (Figure 4Bi). Representative flow-gating demonstrates the prominent OT1 T-cell population within the OT1-injected tumors, while the 2C-injected tumors show a smaller population of 2C T-cells within the tumor (Figure 4Bi). Quantitative analysis of the flow-data revealed a higher amount of total CD8^+^ T-cells within the OT1-injected tumors (*p* = 0.09), and the increase was mostly made up of OT1 cells (*p* < 0.05) (Figure 4Bii). Conversely, the 2C-injected tumors showed a low proportion of 2C T-cells within the CD8^+^ T-cell population infiltrating the tumor. These data demonstrate that OT1 T-cells represented the majority of CD8^+^ T-cells infiltrating the tumors, and reveal antigen-specific expansion and successful disruption of the tumor immune environment.

To determine whether antigen-specific T-cell transfer impacted the broader immune environment of the tumor, we analyzed the infiltration of the major myeloid populations in the tumor (Figure 4Ci). Neutrophils represent the largest single infiltrating immune population in these tumors, and these were significantly decreased in number following the introduction of antigen-specific OT1 T-cells in the biomaterial (*p* < 0.001) (Figure 4Cii). There was no significant difference in the proportion of tumor-associated macrophages (TAM’s) or monocytes (Figure 4Cii), or in the small population of dendritic cells (not shown), though it remains possible that the differentiation of these cells was impacted. These data clearly show that the injection of OT1 T-cell biomaterial brought a major change of the tumor immune environment, with contrasting effects on the proportions of CD8^+^ T-cells and neutrophils, and exhibiting a high infiltration of the injected OT1 T-cells. However, despite these positive changes, T-cell transfer was not sufficient to induce a long-lasting tumor control.

### T-cell biomaterial control of recurrence following surgical resection

**Figure 5:**
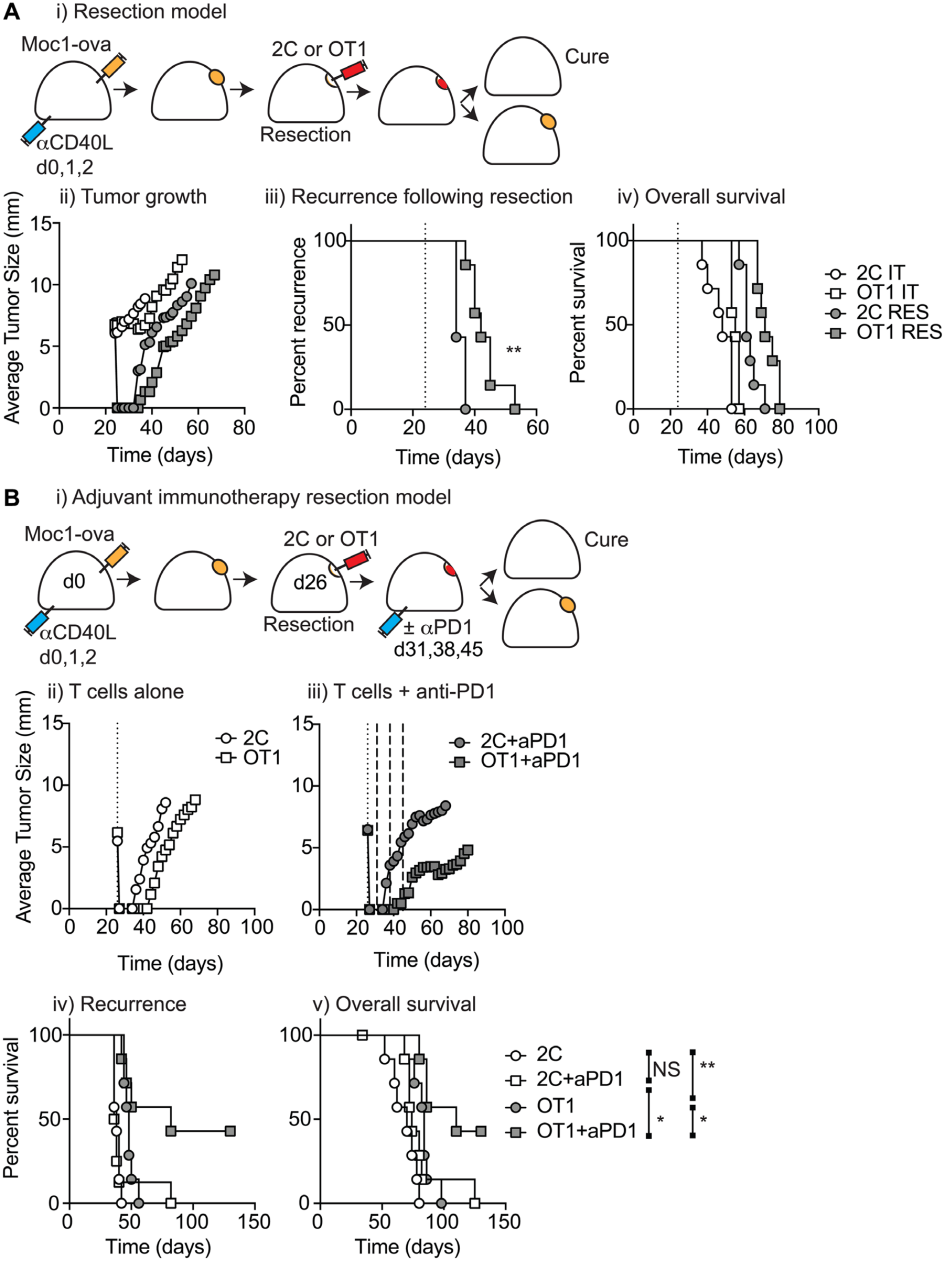
T-cell biomaterial control of recurrence following surgical resection. (**A**) (i) MOC1-ova tumors were established in immune competent C57BL/6 mice and underwent IT administration of tumor-specific OT1 T-cells or non-specific 2C T-cells in 30 μl of Matrigel into the tumor, or subcomplete resection and administration of the same biomaterials into the resection cavity. Graphs show (ii) average tumor size for all groups (iii) recurrence for resection groups, and (iv) overall survival of all treated mice. (**B**) (i) Immune competent mice bearing MOC1-ova tumors underwent surgical resection and administration of T cell biomaterials into the resection cavity as per a). Mice were randomized to receive no further treatment or 3 weekly doses of adjuvant anti-PD1 starting 5d following resection. Graphs show average tumor size for (ii) T-cells alone or (iii) T-cells plus adjuvant anti-PD1. Graphs show (iv) recurrence following resection, and (v) overall survival of treated mice. Abbreviation: NS: not significant. ^*^ *p* < 0.05, ^**^ *p* < 0.01.

Successful T-cell killing of targets can be highly impacted by effector to target ratios, and so may be much more effective in minimal residual disease settings. Our primary goal is to develop approaches that prevent local recurrence following surgical resection, and biomaterial delivery of T-cells may be optimal to drive locoregional immunity via treatment of the resection cavity. To model this, we made use of a tumor resection model that results in consistent local recurrence that is treatable via immunotherapy [9, 12]. Moc1-ova tumors were established in immune-competent C57BL/6 mice and mice that were randomized to no-surgery or subtotal resection of the tumor. Mice were further randomized to receive biomaterials loaded with tumor-specific OT1 T-cells or non-specific 2C T-cells, either delivered intratumorally in the non-resection group as before, or into the resection cavity (Figure 5Ai). Mice were followed for tumor growth, recurrence following surgery, and overall survival. As expected, surgical resection led to a tumor-free period followed by rapid local recurrence (Figure 5Aii). Administration of antigen-specific T-cells into the resection cavity significantly delayed recurrence (*p* < 0.01) (Figure 5Aiii), and led to a significant increase in overall survival (*p* < 0.01). However, all tumors recurred and the combination of T-cell transfer and tumor resection, while superior to direct injection into unresected tumors, did not result in tumor cure (Figure 5Aiv). Therefore, despite the benefit in locally administering T-cell biomaterial into the resection cavity, all the groups eventually failed.

In tumors, exhaustion of tumor-specific T-cells is commonly observed and can be overcome through the administration of checkpoint inhibitors. To determine whether failure of local control is a result of T-cell exhaustion, MOC1-ova tumors were established in the same manner and all mice underwent subtotal resection of the tumor followed by immediate application of tumor-specific or non-specific T-cell biomaterials into the resection cavity. Mice were also randomized to receive 3 doses of 250 μg PD1-antibody at days 5, 12, and 19 after resection (Figure 5B). As before, antigen-specific T-cell transfer significantly delayed tumor recurrence (OT1 vs. 2C *p* < 0.01), though all mice recurred (Figure 5Bii–v). Anti-PD1 treatment did not impact recurrence or survival in the absence of antigen-specific T-cell transfer (2C vs. 2C+anti-PD1, median survival 70 vs. 74 days, respectively), consistent with data showing that pre-existing immune responses in tumors are essential to the success of checkpoint inhibitors [20, 21]. Importantly, administration of anti-PD1 significantly delayed recurrence in mice treated with antigen-specific T-cells in the biomaterial (OT1+anti-PD1 median survival 110 days, *p* < 0.05 vs. 2C+anti-PD1 median survival 74 days, *p* < 0.05 vs. OT1 median survival 84 days), and this resulted in long-term tumor cures (Figure 5Biv–v). These data emphasize that biomaterial delivery of tumor antigen-specific T-cells significantly delays local recurrence and that the addition of adjuvant PD1-blockade can prevent local recurrence and permit tumor cures. We propose that this is a potential intervention for HNSCC patients with poor pre-existing anti-tumor immunity with a high risk of local recurrence, who are currently poorly served by existing treatment options.

## DISCUSSION

Our data demonstrate that local administration of T-cells into a biomaterial preserves T-cell function *in-vitro* and *in-vivo*. By using a biomaterial that forms to the resection cavity we can deliver T-cells locally following surgery, retain T-cell function, and delay tumor recurrence. We show that adjuvant anti-PD1 is ineffective following surgery in mice that lack pre-existing anti-tumor immunity, but tumor antigen-specific T-cell transfer combines with anti-PD1 to prevent local recurrence and cure a subset of mice.

Our model uses treatment with anti-CD40L at tumor implantation to eliminate pre-existing immune responses to the tumor. Injection of cancer cells in suspension has long been known to generate anti-tumor immune responses [22, 23], and this can lead to spontaneous rejection of highly immunogenic tumors [23, 24]. We recently demonstrated that anti-CD40L prevented the generation of resident memory phenotype T-cells [19], which are highly impactful to the outcome of patients with HNSCC following treatment conventional therapies [25]. By using anti-CD40L at tumor implantation, we generate tumors with low numbers of T-resident memory CD8^+^ T-cells, and we would expect a poor response to checkpoint inhibitors in these animals as observed in patients [26]. Importantly, we demonstrate that local adoptive transfer of tumor antigen-specific T-cells can overcome the lack of pre-existing immunity and allow effective combination with checkpoint inhibitors to control residual disease. Expression of the SIINFEKL peptide increases the baseline immunogenicity of the Moc1 cell line [27], which can result in long-term antigen specific protective immunity. However, while mice cured of tumors by implantation of OT1 T-cells and PD1 blockade remained tumor-free long-term, we did not test whether these mice could reject rechallenge with the Moc1-ova or the parental Moc1 cell line. It would be valuable to know that the therapy results in long-term survival of tumor-specific T-cells to ensure long-term protection against recurrence or control of previously undetected micrometastases.

Currently, adoptive T-cell therapy for HNSCC and other cancers is administered intravenously, and this is critical for patients with metastatic cancer since the tumors may be widespread. However, systemic delivery of T-cells still necessitates multiple steps for functional tumor control. Adoptive transfer of T-cells requires *in-vivo* antigen-specific expansion following transfer [6, 7], which may present a problem following surgical resection. We have previously demonstrated that the capacity for T-cell expansion in tumor-draining lymph nodes is rapidly lost following resection [9]. This can be overcome through a combination of lymphodepletion prior to transfer which drives homeostatic T-cell expansion [28], and post-transfer antigen-specific vaccination [29]. The combination of these treatments results in the expansion of memory phenotype T-cells that are more effective in tumor control [30]. We propose that locoregional therapy to the resection site can take advantage of residual tumor antigen to permit expansion of both effector and memory cells without the need to lymphodeplete the patient or prepare a patient-specific vaccine. Importantly, we demonstrate that tumor-specific cells expand in number in the peripheral circulation and accumulate at the tumor, indicating that lymphocyte recirculation is fully functional when T-cells are delivered to the resection site. Recirculation is critical to effectively detect tumors that may additionally be present at distant sites, and sustain systemic anti-tumor immunity [10].

Biomaterial delivery is logical for delivery to the resection site, which may be highly disrupted and highly variable between patients. Our approach used a simple biomaterial for rapid release of the T-cells; however, this approach can be refined to include additional agents to support T-cell activation and expansion [16, 17] and modified to control T-cell release kinetics. Importantly, when T-cells were delivered locally in a biomaterial, there was no negative impact on their numbers in the systemic circulation, suggesting that this platform has the potential to provide systemic immunosurveillance for distant disease. Further studies are necessary to map the activation and distribution pattern of the T-cells to understand the effect of local delivery on activation in the local site versus draining lymphatics, and the effect of this therapy on recirculation kinetics [10]. In HNSCC, resection of the advanced tumor, followed by adjuvant therapy, will fail in approximately half of the patients, with 20-30% of patients failing locally [31, 32]. Therefore, locoregional T-cell delivery may be an effective tool to generate improved anti-tumor immunosurveillance both within and outside the primary tumor site – an important feature for HNSCC management.

In our preclinical model, we were able to source large numbers of tumor antigen-specific T-cells from congenic TCR transgenic mice. In patients, sourcing these cells will be a much more significant problem. Traditionally, T-cells for adoptive transfer are generated by cytokine-driven *in-vitro* expansion using tumor fragments obtained from a resected tumor [33]. In patients with widely metastatic melanoma a surgically accessible tumor can provide the tissue to expand tumor-specific T-cells, but there may not be equivalent opportunities that fit with current surgical management of HNSCC. Recent advancements in tumor antigen identification using genomic analysis of patient-specific tumor mutations have permitted *in-vitro* expansion of highly selective T-cell clones for adoptive transfer [34–36], though at present these cells are still expanded from surgical material. If we are to administer these T-cells into the resection cavity immediately following surgery, alternative sources of tumor-specific T-cells will be necessary.

Together, these data demonstrate a potential therapy for patients with poorly infiltrated tumors with a high risk of recurrence following surgical resection. Further work is needed to develop a biomaterial for clinical translation. A range of potential biomaterial options are available for direct depot injection of immunotherapies alone, or in surgical settings (reviewed in [11, 37, 38]). In addition, it will be valuable to evaluate additional components to support T-cells and control the local inflammatory environment to maximize T-cell control of residual disease.

## MATERIALS AND METHODS

### Ethics

All animal protocols were approved by the Earle A. Chiles Research Institute IACUC (Animal Welfare Assurance No. A3913-01).

### Animals and cell-lines

6 to 8-week-old female C57BL/6 mice were obtained from The Jackson Laboratory. Survival experiments were performed with 6–8 mice per experimental group, and mechanistic experiments with 4-5 mice per group. The Moc1 murine HNSCC cell-line was kindly provided by Dr. Uppaluri (Dana Faber Cancer Institute, MA) [39]. A plasmid encoding a fusion protein of GFP with a C-terminal OVA_257–264_ (SIINFEKL) tag to ensure non-secreted cytoplasmic expression of the model antigen has been previously described [19]. Moc1 cells were transfected with the GFP-SIINFEKL construct and GFP^+^ cells were sorted by flow cytometry to generate a stable GFP^+^ population. Presentation of SIINFEKL was confirmed using a B3Z T-cell assay [40], with Moc1-ova or control cells seeded with B3Z T-cells, and antigen-specific recognition confirmed by β-gal assay. Species identity checks on these murine cell lines were performed with murine-specific MHC antibodies, and were tested for contamination within the past 6 months using a Mycoplasma Detection Kit (SouthernBiotech, Birmingham, Alabama). 2C-mice transgenic for a TCR recognizing the model antigen SIYRYYGL were kindly provided by Dr. Gajewski (University of Chicago, Chicago, IL). OT1-transgenic mice were gifted by Dr. Redmond (Earle A. Chiles Research Institute). For *in-vitro* culture or adoptive transfer, spleens from these mice were harvested and CD8^+^ T-cells were isolated using EasySep kit (Stemcell Technologies, Vancouver, Canada).

### Antibodies and reagents

Flow-cytometry antibodies included CD3e-PE, PD1-BV605, CD45-BV786 (BD-Biosciences), CD90.1-FITC, CD62L-PECy7, CD45.1-APC, CD45.1-PE (eBioscience), CD45.2-Alexa700, CD-8α-Percp-Cy5.5, CD4-FITC, CD103-APC, CD24-APCcy7 (Invitrogen), CD44-APCy7, CD4-BV421, F4/80-PercpCy5.5, CD39-PECy7, PDL1-PE, CD90.2-Alexa 700, MHCII-BV421, CD11b-BV650, Ly6C-BV711 (Biolegend), CD8α-PE-Texas-red (Life Technologies, Carlsbad, CA, USA). Blocking PD-1 antibody (clone RMP1-14, BioXCell, Branford, CT, USA) was administered systemically by intraperitoneal injections of 250 μg [41]. Blocking anti-CD40L (clone MR1, BioXCell) was administered systemically by intraperitoneal injections of 250 μg [19].

### *In-vitro* T-cell viability and function

Purified CD8^+^ T-cells from transgenic 2C or OT1 splenocytes were loaded into Matrigel (Corning Inc., Corning, NY, USA) to form 50 μl pellets, each containing 1.5 × 10^5^ CD8^+^ T-cells. The pellets were seeded to Matrigel-coated plates and allowed to solidify at 37°C for 30 minutes before submersion in media. The number and viability of cells were determined using ViaCount reagents (Luminex) according to manufacturer instructions. Viable and non-viable cells are distinguished using DNA-binding dyes and read on a Guava easyCyte (Luminex). The absolute cell numbers and percent viable cells were used to evaluate the release of viable T cells from Matrigel pellets. At each time-point 10 wells were analyzed for 10 pellets and 10 supernatants. To monitor antigen-specific control of cancer-cells, tumor cells were plated, and purified CD8^+^ T-cells from transgenic 2C or OT1 splenocytes were loaded into Matrigel to form 50 μl pellets, each containing 1.5 × 10^5^ CD8^+^ T-cells as described. The pellets were seeded to cancer-cells coated plates and imaged using Incucyte (Sartorius, Goettingen, Germany).

### *In-vivo* models

Tumors were inoculated subcutaneously into immune-competent female mice at a dose of 5 × 10^6^ Moc1-ova cells, and three doses of 100 μg anti-CD40L were administered at days 0,1, and 2 to eliminate the immune response at tumor challenge and establish poorly infiltrated tumors [19]. Prior studies have demonstrated that all effects of anti-CD40L are lost within 14 days [19]. Tumors were allowed to develop to 6–8 mm in diameter over 20–30 days, at which point the mice were administered with 1 × 10^6^ T-cells. In the intravenous (IV) model, OT1 or 2C cells were delivered in 100 μl of PBS; in the intratumoral (IT) model, OT1 or 2C cells were suspended in Matrigel mixed 1:1 with PBS and a total volume of 30 μl was injected intratumoraly; in the subtotal resection model, following surgical skin prep, an incision was made over the tumor and the encapsulated tumor was mechanically detached from the skin. The tumor capsule attached to the underlying fascia was left in place and the upper portion of the tumor resected, leaving 2–2.5 mm depth of tumor in the resection cavity, and 30 μl of the above-mentioned mixture with 1 × 10^6^ T-cells was placed and allowed to solidify in the surgical site. Mice that were randomized into adjuvant immunotherapy group received three doses of intraperitoneal 250 μg PD-1 antibody in 100 μl of PBS, 5, 12, and 19 days post-operatively.

### Flow-cytometry

For analysis of circulating tumor-specific T-cells, whole-blood samples were collected at designated time-points, and whole blood was surface stained for phenotypic markers, and counting beads were included to permit quantitative analysis of circulating cells [19]. The mixture of antibodies included CD3, CD4, CD8α, CD44, CD45.1, CD45.2, CD62L, and CD90.1, to distinguish circulating CD3^+^CD8^+^CD90.1^+^ OT1 T-cells and CD3^+^CD8^+^CD45.1^+^ 2C T-cells from host-cells.

For analysis of tumor-infiltrating tumor-specific T-cells, tumors grown in C57BL/6 mice were harvested 7 days after treatment, and single-cell suspensions were prepared by using a gentleMACS dissociator (Miltenyi Biotech, Bergisch Gladbach, Germany), followed by agitation in digest solution (250 U/mL collagenase, 30 U/mL DNase, 5 mM CaCl_2_, 5% FBS in HBSS in PBS) for 30 minutes at 37°C. The digest was filtered through 100 μm strainer to remove macroscopic debris. Cells were surface-stained with the phenotypic markers CD45.1, CD45.2, CD90.2, CD103, CD4, CD8, CD11b, MHCII, Ly6C, CD11b, CD24, F4/80, and a viability dye to distinguish live CD11b^+^MHCII^+^Ly6C^-^F4/80^+^ macrophages, CD11b^+^MHCII^-^Ly6C^+^ neutrophils, CD11b^+^F4/80^-^Ly6C^+^ monocytic cells, and CD11b^+^MHCII^+^Ly6C^-^CD24^+^ dendritic cells [42], as well as the adoptively transferred OT1 and 2C T-cells as described above. Samples were run on a BDIS Fortessa SORP or BDIS LSRII SORP (BD Biosciences) and analyzed using FloJo (Tree-Star, Ashland, OR, USA). Infiltrating cell proportions are shown as a percentage of live cells to control for varying degrees of overall infiltration in the tumors and permit direct comparisons between groups.

### Multiplex IHC

Tumors grown in C57BL/6 mice were harvested 7 days following treatment, zinc-fixed, sectioned and prepared as previously described [43]. Primary antibodies were anti-CD3 (SP7, Abcam, Cambridge, UK), anti-CD8α (4SM15, eBioscience), anti-FoxP3 (FJK-16s, Invitrogen, Carlsbad, CA, USA), anti-PD-L1 (D5V3B, Cell-Signaling Technology, Danvers, MA, USA), and DAPI (Perkin Elmer). Anti-Rat HRP (mouse adsorbed) or Anti-Rabbit HRP secondary antibodies (Vector Laboratories) were used prior to tyramide staining with Opal 7-Color Automation IHC Kit (690 for CD3, 570 for PD-L1, 620 for CD8α, and 520 for FoxP3). Slides were scanned with Vectra Polaris (Perkin Elmer) and analyzed using QuPath 0.2.0-m4 [44].

### Statistical analysis

Data were analyzed and graphed using Prism (GraphPad Software, La-Jolla, CA, USA). Individual data sets were compared using the two-tailed unpaired Student *t*-test; overall survival of groups was compared using the log-rank test for differences in Kaplan–Meier survival curves.

## Abbreviations

HNSCC: head and neck squamous-cell carcinoma
PD1: programmed cell death protein 1
MOC: mouse oral cancer
ova: ovalbumin
IT: intratumoral
IV: intravenous
IHC: immunohistochemistry
TAM: tumor-associated macrophages
TCR: T-cell receptor
